# ‘God is always on my side’: internal and external predictors of workplace bullying targets’ help-seeking behavior in a religious context

**DOI:** 10.3389/fpsyg.2024.1481718

**Published:** 2024-11-26

**Authors:** Mykolas Deikus, Jolita Vveinhardt

**Affiliations:** ^1^Department of Theology, Faculty of Catholic Theology, Vytautas Magnus University, Kaunas, Lithuania; ^2^Vytautas Kavolis Interdisciplinary Research Institute, Vytautas Magnus University, Kaunas, Lithuania

**Keywords:** workplace bullying, religious coping, theory of planned behavior, subjective norms, help-seeking behavior, religious community support, mixed methods

## Abstract

Workplace bullying is a pervasive issue that affects millions of individuals worldwide, leading to severe psychological and social consequences. This study examines the factors influencing the decisions of religious individuals who have experienced workplace bullying, with an explicit focus on their choice to seek help from their religious community. The study involved respondents from various religious groups, most of whom were Roman Catholic. The research employs a cross-sectional design, integrating quantitative analysis with qualitative insights. Data from 1,231 respondents were analyzed via descriptive statistics, correlation, regression, and content analysis methods. The main coping strategies identified were self-coping and seeking help within a close, trusted environment. Attitudes toward the religious organization (subjective norms) consistently predicted help-seeking decisions more accurately than access to religious counseling (perceived behavioral control) or personal values. This research provides one of the first empirical insights into how religious communities can actively mitigate the psychological impact of workplace bullying, offering a novel perspective on the intersection of faith and mental health. The findings suggest that religious organizations could be crucial in supporting victims by enhancing outreach and counseling services, contributing to a holistic approach to workplace well-being. These findings have significant implications for religious communities, highlighting their potential to support their members in times of active distress.

## 1 Introduction

Intervention in workplace bullying is a complex task, the solution of which often depends on answering the question of what reasons prevent both men and women from seeking help in a timely manner (MacIntosh et al., 2011; O’Donnell and MacIntosh, 2016). This intricate problem has been the focus of research for many years, with solutions often directed toward individual (e.g., Chomczyński, 2020; Jóhannsdóttir and Ólafsson, 2004; Salin, 2021; Seto et al., 2020) and organizational environment-related (e.g., Dean et al., 2021; Mulder et al., 2016; Seto et al., 2020; Petrie et al., 2022; Tarablus and Yablon, 2023) factors. The results of these studies provide a deeper understanding of victim behavior and the potential removal of barriers to timely access to effective help-seeking.

Understanding the factors that encourage or inhibit help-seeking is vital because organizational changes, strategies and training programs targeting individual employees may not necessarily achieve the desired effect (Petrie et al., 2022). This may be explained in part by the specificity of the phenomenon itself. Workplace bullying differs from other conflicts in terms of the frequency of incidents, the negative impact of social behavior, power imbalances, the durability of the process and the intentions of persecutors (Baillien et al., 2017) and thus causes severe and multidimensional harm (Sansone and Sansone, 2015). In this context, research shows that workplace bullying reduces workers’ efforts to seek help and that workers themselves perceive more barriers to help than those who are less affected (e.g., Seto et al., 2020; Tarablus and Yablon, 2023). These findings encourage us to delve further into the issue, look for avenues that have not yet been tested, and take a broader view by evaluating the work of researchers studying the issue of assisting survivors of violence outside the workplace.

These pieces of research are crucial in addressing a pressing issue in the workplace and can significantly contribute to the fields of psychology, sociology, and organizational behavior. That is, in addition to providing knowledge of individuals’ experiences and motives for their behavior, it explains the dynamics of social interactions and power in the work environment, can contribute to the search for ways to improve the prevention of workplace bullying and use more efficient employee support strategies.

One promising yet largely unexplored area in workplace bullying is the role of religious spirituality, which, in the context of this study, is understood as a combination of spiritual practices (e.g., prayer, meditation, reading religious texts) and religious faith in order for the person to achieve a connection with transcendence through religious faith and rituals (Jastrzębski, 2022). Research on religiosity, conducted in different cultural contexts, reveal the complex and potentially beneficial role of religious coping in the help-seeking attitudes of victims of violence, such as in overcoming self-stigmatization (Locke and Kim, 2024; Deikus and Vveinhardt, 2024). While Seto et al. (2020) did not specifically examine the religiosity of workplace bullying targets, they suggested that reducing stigma and other barriers, thereby increasing the availability and attractiveness of evidence-based support, could significantly aid workers in coping with the effects of workplace bullying in their workplace. This research opens a new avenue for understanding and potentially addressing workplace bullying, offering hope for improved interventions and support systems.

Coping with workplace bullying has been found to involve existentialist issues (D’Cruz and Noronha, 2013), the answers to which can be found in religion and religious counseling for religious individuals (Lee et al., 2020). In addition to support from friends and family, religious beliefs and the answers found from their perspective play essential roles in making sense of suffering or providing comfort (D’Cruz and Noronha, 2012; Mokhtar and Kamaluddin, 2021). Nevertheless, other studies show that religiosity works as a preventive measure in adolescents’ relationships (Hong et al., 2024) and that a higher religiosity of an individual decreases the likelihood of a worker responding to bullying with negative actions (De Clercq, 2022). However, it still needs to be made clear which response strategy religious employees choose to respond to workplace bullying and what factors are essential for religious individuals in this context when seeking counseling. This gap in our understanding presents an intriguing area for further exploration and research. What is important here is the extent to which the reactions of the targets of workplace bullying depend on the cultural context and the degree to which the results can be applied. For example, Escartín et al. (2010) has found that employees’ perceptions of workplace bullying do not vary substantially across regions of the world (Southern Europe and Central America, where the majority of the population is Catholic), which has some positive implications for designing and implementation of various coping strategies in such parts of the world where little research has been done. A subsequent review of research by Nielsen and Einarsen (2018) has shown that the link between bullying and subsequent mental health problems is particularly strong, since this has been identified in both general and occupational samples as well as across countries and cultures. In addition, the research review conducted by Karatuna et al. (2020) has demonstrated that both the consequences of workplace bullying across cultures coincide and reactions can be similar. For example, emotion-focused coping was more common in almost all cultures, while problem-focused coping strategies were highlighted in the studies conducted in relatively more assertive cultures (e.g., Eastern Europe, Anglo and the Middle East, where religious contexts differ strongly).

According to Lefevor et al. (2022), the role of religiosity in help-seeking is well explained by the theory of planned behavior, which takes into account a person’s attitudes, intentions, and perceived ability to engage in certain behaviors or social norms. As different help-seeking strategies (e.g., within the family, among friends, in public institutions) are influenced by religious institutions, both directly through the representatives of these institutions and indirectly through values and teachings, Chaudhry and Cattaneo (2023) argue that they all need to be included in the research on help-seeking. In this context, it is important to appreciate that trusted religious community representatives can act as intermediaries who provide support and advocacy and encourage referrals to other professionals. Therefore, given the lack of research in this area, this exploratory study aims to identify the factors that influence the decisions of targets of workplace bullying to seek help from leaders of their religious community or religious counselors. Specifically, three questions are posed. Q1: What response strategies are chosen by religious workers in response to workplace bullying? Q2: How do different experiences of relationships with co-workers relate to motives for seeking religious counseling? Third, how do internal and external circumstances influence motives for seeking religious counseling (Q3)?

This exploratory research is partly based on the principles of the theory of planned behavior (TPB), which helps understand human behavior under specific circumstances (Ajzen, 1991). In the context of this study, the TPB is designed to analyze behavioral intentions, subjective norms and perceived behavioral control of targets of workplace bullying in their help-seeking process. These core elements of the TPB helped to structure the questionnaire and to identify factors that encourage or hinder help-seeking. Subjective norms are particularly important in analyzing the relations of targets with religious communities, because victims’ behavior was directly related to their attitude toward the religious community and its representatives’ support. This way, the TPB served to operationalize variables and explain how they interact in the religious context of help-seeking. Ajzen (2020) noted that beliefs and subjective norms may vary from situation to situation or from population to population; thus, he did not propose a universal questionnaire but suggested flexibility and the use of relevant variables for prediction. The exploratory study, therefore, uses a specially designed questionnaire and compares three groups of individuals: Group A, those who have experienced unsystematic assaults; Group B, those who have experienced systematic assaults; and Group C, those who have not experienced any negative behavior (see below for a detailed description).

A comparison between these groups allows us to highlight how different levels of bullying affect people’s decisions to seek help and to analyze the factors that encourage or hinder help-seeking. A distinction is usually made between unsystematic, isolated conflicts (group A in this study) and systematic, prolomged persecution, which is associated with significantly more severe consequences (group B) (Budnik, 2020; Leymann, 1990; Tatar and Yüksel, 2019). The third group was distinguished for comparison with the responses of people who did not experience any negative forms of behavior.

The results of the study by Seto et al. (2020) revealed that targets of workplace bullying perceived barriers such as not knowing where to obtain medical or counseling help, anxiety about confidentiality, the cost of services, etc. Personal values and attitudes about the extent to which the religious community is potentially willing to help were also highlighted. According to Ajzen (2020), the actual experience of behavior and the reactions of significant others are important; therefore, the existing experience of the relationship with the religious community and the reactions of significant others (relatives, religious leaders) were additionally assessed. These factors may help or hinder behavior in the context of perceived behavioral control.

The article is thus divided into several parts. First, on the basis of a semi systematic literature review approach, it theoretically defines the construct of workplace bullying and briefly discusses the factors that hinder or encourage help-seeking. Then, the research methods, results and interpretations are presented. Finally, the results of the study are discussed, and the main conclusions are presented. The most important factors that promote or inhibit help-seeking are related to the intensity of the experience, the values of the target of workplace bullying and attitudes toward religious organizations.

## 2 Theoretical framework

*Workplace bullying*, as Budnik (2020) defines it, is a serious issue that arises from the complex interplay between a group and an individual. According to Nielsen et al. (2019), “workplace bullying is not about single episodes of conflict or harassment at the workplace, but a form of persistent abuse.” It involves the systematic use of hostile and unethical actions directed against one individual, with these acts being repeated frequently and continuing over a long period of time. The seriousness of this problem is highlighted by recurrent and persistent negative behavior, which often continues over a long period of time, sometimes lasting 6 months or more, and causes severe psychological, psychosomatic and social suffering (Budnik, 2020; Leymann, 1990). The impact of such prolonged suffering on an individual’s professional performance is significant, leading to decreased productivity, increased absenteeism, and even resignation from the job. Therefore, it is imperative for all stakeholders to unite and commit to addressing workplace bullying collectively.

However, a review of the use of the term workplace bullying in different contexts (Saunders et al., 2007) lacked a clear consensus on how often and for how long a negative behavior has to persist before it is labeled bullying. No clearer consensus seems to have emerged over time. For example, in Nielsen et al.'s (2019) study, bullying was categorized as negative behavior that was repetitive (nonisolated), and one of the parties had difficulty defending themselves (unequal power of the conflicting parties). In this study, we apply the term workplace bullying to conflicts that recur at least once a week and last at least six months (based on Leymann, 1990). This is done to distinguish conflicts that are intensely escalated from conflicts that are sporadic, repeated for a limited period of time, or take place over a long period of time but in an unsystematic way (discussed in more detail in the presentation of the questionnaire).

*Religious counseling* is one of the sources of help in dealing with a wide range of violent and emotional problems (Amstadter et al., 2008; Chaudhry and Cattaneo, 2023; Postmus et al., 2009). In accordance with the definition proposed by Worthington (1986), religious counseling is understood as counseling that is based on the content of a specific religion or that takes place in an explicitly religious context (e.g., in a pastor’s church). Although religious counseling is most often provided by clergy, laypeople with special training may also be counselors. In the Christian context, Kim (2003, p. 50) distinguishes pastoral counseling from ‘Christian counseling proper’. Pastoral counseling is part of the pastoral profession and is understood within the framework of pastoral care. Christian counseling, on the other hand, is similar to general counseling, except in content. It is, therefore, defined as “the process of encountering to liberate people for God and man” (Kim, 2003, p. 54). On the other hand, the nature of pastoral counseling is also changing. The results of a study by Saar et al. (2015) show that the changes are marked by the use of secular language in counseling, the interpretation of events via psychological theories, and the development of treatment plans in light of the psychological changes.

The theory of planned behavior is described as a social cognitive model, which states that behavior is a function of intentions (personal attitudes about behavior and beliefs about what significant others think) and perceived behavioral control (the belief about how easily certain resources can be accessed and the individual’s confidence in their ability to use these resources) (Ajzen, 1991; Conner, 2001; O’Doherty et al., 2016). According to this theory, targets of workplace bullying may face internal or external factors that hinder or encourage them to seek help from a religious counselor.

A study by Seto et al. (2020) revealed that structural barriers, such as not knowing where to obtain medical or counseling help, concerns about confidentiality, and the cost of services, were significant factors that hindered individuals from seeking help. In addition, high levels of violence and the associated consequences for the individual reduce efforts to seek help (Tarablus and Yablon, 2023). An assaulted person may interpret his/her situation as sufficiently difficult and complex not to seek help or ways to change it (Chomczyński, 2020). Other research suggests that factors such as others’ perceptions of the problems experienced (Klinefelter et al., 2021), social support or lack thereof (Pezaro et al., 2021), previous experiences of interacting with a helping professional (Genest et al., 2021), and perceived usefulness of the help are important in seeking help from a specialist (O’Donnell and MacIntosh, 2016).

The values and strengths of religiosity and community support play crucial roles in the help-seeking process for individuals who identify with a particular religious group. For instance, religious clients often prefer counselors whose values align with their own values, and the strength of religious commitment is often linked to therapy expectations (Worthington, 1986; Worthington and Sandage, 2001). Moreover, the decisions of victims to seek help are positively influenced by the support of their family or religious communities (Chaudhry and Cattaneo, 2023).

## 3 Methods

### 3.1 Design

This study is based on a cross-sectional quantitative study design combined with elements of qualitative research. This choice was determined by the fact that the goal was not only to analyze quantitative data on help-seeking behavior but also to gain a deeper qualitative understanding of subjects’ choices. Mixed methods provide an opportunity to expand interpretations of research findings, as quantitative data reveal general trends, while qualitative data provide a deeper understanding of personal experiences of research participants (Creswell and Plano Clark, 2017).

The said methods were applied simultaneously, in order to collect both quantitative and qualitative data at once, which allowed simultaneous analysis of various help-seeking processes. This way, it was possible to compare and combine the results, which increases the possibilities to revise and supplement them (Creswell and Plano Clark, 2017; Morse, 2010). The combination of methods adds value to the study, since this allows identifying not only general trends in help-seeking but also gaining a deeper insight into invovement of religious communities in the support provision process, which could be missed when conducting the quantitative analysis alone.

### 3.2 Sampling

Snowball sampling was used in this study. Although this method is commonly used in qualitative research, in quantitative research, it is used to study hard-to-reach populations (Mweshi and Sakyi, 2020). Survivors of workplace bullying are particularly vulnerable and experience anxiety, shame, guilt, and stigma (Leymann, 1990; O’Donnell and MacIntosh, 2016; Seto et al., 2020), and recommendations from people within the religious community network (e.g., community leaders, clergy, or survivors participating in the study) are useful for building trust in the researchers and recruiting participants. According to Baltar and Brunet (2012), the limitations of the traditional snowballing approach are reduced by sampling, which involves an initial ethnographic assessment to identify networks that may exist in a given population. Subgroups are then considered a cluster sample, and coverage bias is reduced, resulting in increased representativeness. The sample size is based on the number of employed persons. In 2023, 1.44 million registered workers were registered in the whole country.^1^ At least 385 respondents needed to be interviewed to ensure a confidence level of 95% (±5% margin of error).

### 3.3 Ethical considerations

The research was approved by the Ethics Committee of the Faculty of Economics and Management of Vytautas Magnus University (permission No 2023--11--09, 2023–11/1). Potential participants were informed in writing about the purpose of the study, the guarantees of anonymity, confidentiality, free-will participation, and the right to withdraw from the questionnaire at any time, which allowed them to freely provide informed consent to participate in the study.

### 3.4 Instrument

The survey uses a 40-item Likert-type scale, “Motives of Persons Aggrieved at Work for Seeking Spiritual Assistance (MP-SSA-40),” adapted to examine factors that influence decisions to seek religious help (Vveinhardt and Deikus, 2021). The questionnaire consisted of scales measuring *the experience of hostile actions by coworkers at work*, indicating situational factors (Leymann, 1996; Zapf et al., 2020) (e.g., “They shouted at me loudly, used offensive words”); *knowledge of the availability of a religious counselor* in relation to perceived behavioral control (Vokurka et al., 2002) (e.g., “This was talked about in religious community meetings”). Other scales are designed considering the model of the individual-church relations, proposed by Pessi (2013), which consists of the interaction of experience, values (both expectations related to the church and personal values related to the church), truth (foundations for reflection, an offer of religious activity, clear positions and space for individuality) and authenticity, which manifests itself in all three elements. Combining the insights of this model with the questionnaires of Pargament (2003a,b), Idler (2003), Krause (2003), the following scales were distinguished: respondents’ *values*, reflecting attitudes toward the actions (e.g., *“It is important to me that people help without expecting to be rewarded”*), *attitudes toward the religious organization*, which were related to subjective norms (e.g., “I feel that I belong to my religious community and I am welcome there”), and motives for seeking religious counseling or factors that encourage or inhibit seeking a counselor (e.g., “It is important for me to be assured confidentiality”). A 5-point Likert scale was used to measure the strength of agreement or disagreement with the statement (*Strongly disagree, Disagree, Neither agree nor disagree, Agree, Strongly agree*).

The respondents were also asked to indicate their preferred problem-solving strategies (e.g., contacting a psychologist, a company manager, a religious counselor, etc.) (e.g., “I turned to a psychologist,” “I did not contact anyone because I did not believe anyone could help”). These statements are formulated based on (Kohnke and Winiarski, 2019; Litzcke and Schuh, 2005) and presented using a nominal forced-choice scale.

The scale for measuring hostile behavior consists of two parts. The first part asked respondents to review statements that corresponded to their personal experiences, whereas the second part allowed respondents to be divided into three groups. Group A (those who had experienced unsystematic assaults) included those who had experienced at least one of the hostile acts listed above but less than once a week. Group B (workplace bullying) included people who had experienced at least one of the listed acts at least once a week for at least 6 months. Finally, Group C consisted of respondents who had not experienced any negative behavior. The demographic data included gender and age, and the respondents were asked to indicate whether they believed in God and which religious denomination they belonged to.

### 3.5 Data collection

First, a baseline group was established. As both researchers provide free counseling to employees affected by workplace bullying, they were contacted and asked to complete an online questionnaire and share it with their acquaintances who had similar experiences. Representatives of religious communities and clergy registered in Lithuania were also contacted. They were informed of the purpose of the study and asked to mediate by passing on information about the study to their community members and forwarding the link to the electronic questionnaire via e-mail. The questionnaire was published on an online website and was completed by 1,231 respondents. No incomplete questionnaires were received (a function was used to prevent the submission of incomplete questionnaires).

### 3.6 Reliability analysis

After performing the analysis of the research instrument, high values of Cronbah’s alpha and Spearman-Brown coefficients were obtained. Compared to other coefficients, the relatively lowest values were obtained when measuring *the experience of hostile actions by coworkers at work* (*α* = 0.85, *r*_SB_ = 0.80), while the highest ones were obtained for the scale *knowing the availability of a religious counselor* (*α* = 0.91, *r*_SB_ = 0.87). The indicators of the reliability analysis of the remaining scales were similar: *personal values* (*α* = 0.90, *r*_SB_ = 0.88), *attitudes toward a religious organization* (*α* = 0.90, *r*_SB_ = 0.84), and *reasons (motives) for seeking religious counseling* (*α* = 0.90, *r*_SB_ = 0.90).

### 3.7 Statistical analysis

IBM SPSS 25 software was used for data analysis. Descriptive statistics were used to find answers to Q1 and Q2. To answer Q3, a linear regression model was constructed. The regression analysis was performed to determine statistical relations between main variables and to evaluate their influence on the dependent variable. This method was chosen because of its ability to predict behavior, taking into account the interaction of variables (Field, 2013). Reliability of the regression model was tested considering assumptions such as normality, multicollinearity and heteroscedasticity, seeking to ensure the validity of the results.

### 3.8 Qualitative data collection and analysis

This method was chosen because of its ability to systematically analyze qualitative data and identify recurring patterns, as recommended by Krippendorff (2018) and Hsieh and Shannon (2005). This approach helps to identify key themes and connections that are important to achieving the purpose of the research.

Qualitative data were collected using one open-ended question that was included in the questionnaire. Respondents were asked to share their experiences related to seeking help and to describe the coping resources they used. The collected qualitative data were analyzed using the content analysis to identify key themes related to the religious resources used, which were effective or ineffective. This method allowed us to gain new insights into the methods of religious support used by the studied population.

### 3.9 Data integration

Quantitative and qualitative data were integrated in the final analysis. Quantitative results helped identify general trends related to help-seeking, while the qualitative data analysis provided a better understanding of the behavior of religious targets of workplace bullying.

## 4 Results

### 4.1 Interpersonal experiences

The majority of respondents were women (64.9%), while more than half were working people aged 21–30 years and 31–40 years (38.1 and 22.8%, respectively). Seven out of ten (70.1%) identified themselves as belonging to the Roman Catholic community, which is broadly in line with trends from the last national census.^2^ The “Other” category includes respondents who did not indicate which community they belong to, as well as individuals who identified themselves as Buddhists, Jehovah’s Witnesses, Pagans, and followers of Krishna. The distribution of respondents by religious community is presented in Table 1.

**Table 1:** Distribution of respondents by religious community.

Belongs to a religious community	%
Roman Catholic	70.1
Evangelical Lutheran	6.9
Evangelical Reformed	0.5
Evangelical Baptist	0.6
Evangelical Methodist	0.1
Orthodox	1.1
Orthodox Old Believers	0.6
Greek Orthodox Catholic	0.6
Other	19.5

According to experiences of interpersonal relationships with co-workers, the largest group consisted of individuals who had not been harmed in the work environment (Group C: 52.9%). A total of 31.7% of the respondents were in Group A (experienced unsystematic assault), and 15.4% were in Group B (experienced workplace bullying).

### 4.2 Coping strategies

When examining how religious survivors cope with their experiences of workplace bullying (Q1), approximately three in ten took independent action, which led to the end of workplace bullying, and approximately two in ten were willing to endure workplace bullying because they did not believe that anyone could help. The solutions chosen by the respondents are shown in Table 2.

**Table 2:** Strategies for coping with hostile coworker behavior.

Solving the problem	%
Solved without the help of others	30.7
Manager support	3.8
Peer support	8.5
Psychological support	2.6
Help from a religious counselor	2.3
Assistance from a lawyer	0.3
Help from relatives	18.5
Passive behavior (did not believe anyone could help)	14.1
Other	19.2

Notably, approximately one quarter sought help outside the organization. In this context, the help of the immediate environment was significant, whereas almost as many people turned to a psychologist and a religious counselor. Among the ‘Other’ respondents, some indicated that they had tried to ignore the actions of perpetrators, changed their attitude toward the negative behaviors, read literature of a psychological nature, sought advice on the internet, consulted a trade union representative or decided to quit their job.

Two types of use of religious support resources (prayer, meditation, Bible reading, hospitality in the religious community, and spiritual accompaniment) emerged among the respondents who shared their experiences (Table 3).

**Table 3:** Types of use of religious resources.

Types	Examples
Individual religious overcoming	“*Presence in the Church and prayer, which helped me to keep going in the hardest times*” “*It has helped that I am a believer, God is always on my side*” “*Relationship with God and His Word in the Bible, I prayed a lot*”
Religious-social coping	“*Going to church helped me calm down and make good decisions*” “*Supported by relatives and friends who were mostly religious*”
Combined overcoming	“*Some help from coworkers, but the most important thing was that I got support and help from my religious community*” “*What helped me the most was spiritual accompaniment and prayer. I also attended specialized training, sought legal help, and am grateful that some of my coworkers and relatives listened to me*.” “*Partly with the help of a psychologist, partly with the help of a clergyman, and finally with the help of the manager*”

*Individual religious overcoming* is characterized by the search for a direct relationship with God through prayer, scripture and sacred space (the church building). *Religious-social coping* was based on the search for social support in a religious community. That is, people who share the same denominational affiliation and therefore the same religious attitudes. *Combined coping*, on the other hand, is distinguished by the variety of resources used (combining religious and secular resources). In this case, religious resources were used as a supportive tool to meet the religious needs of the individual.

### 4.3 Correlation analysis

To determine the answer to Q2, a correlation analysis was performed. That is, the relationships between the factors that encourage or hinder help-seeking and hostile work experiences, personal values, attitudes toward a religious organization and knowledge of the availability of a religious counselor were calculated according to the Hostile Work Experiences group (Table 4).

**Table 4:** Correlation analysis results.

Correlation			Spearman’s correlation coefficient	*p*	Interpretation of the link
Reasons for seeking religious counseling	A *N* = 651	Experiences of hostilities	0.020	0.603	Not statistically significant
		Personal values	0.668**	0.000	Direct, strong, statistically reliable
		Attitudes towards a religious organization	0.711**	0.000	Direct, strong, statistically reliable
		Knowing the availability of a religious counselor	0.552**	0.000	Direct, moderate, statistically reliable
	B *N* = 391	Experiences of hostilities	−0.010	0.843	Not statistically significant
		Personal values	0.677**	0.000	Direct, strong, statistically reliable
		Attitudes towards a religious organization	0.752**	0.000	Direct, strong, statistically reliable
		Knowing the availability of a religious counselor	0.599**	0.000	Direct, moderate, statistically reliable

The correlations were statistically insignificant regardless of whether the individuals were subjected to irregular assaults (Group A) or workplace bullying (Group B). In contrast, the strongest correlations in both experience groups were found between motives for seeking religious counseling and attitudes toward a religious organization (Group A *ρ* = 0.711, *p* < 0.001 and Group B ρ = 0.752, *p* < 0.001). Overall, the correlation coefficients were greater in the workplace bullying group than in the control group, suggesting that the severity of the experience is important in this group. However, the correlation does not indicate the direction of the effect, so further regression analysis was performed.

### 4.4 Regression analysis

To answer the questions of what influences the decision to seek religious counseling and how (Q3), a linear regression model was constructed. The model was based on the respondents’ groups according to their experiences with interpersonal relationships (groups A, B and C) (Table 5).

**Table 5:** Regression model.

	Dependent variable: reasons for seeking religious counseling					
*Group*	*R*	*R^2^*	*R^2^* Correction	*F*	*p*	Cohen’s *f^2^*
*A*	0.754	0.569	0.567	284.699	0.0001	1.320
*B*	0.791	0.625	0.622	215.322	0.0001	1.667
*C*	0.756	0.571	0.564	82.038	0.0001	1.331

Independent variables		Nonstandardized Beta coefficient	Standardized Beta coefficient	*t*	*p*	Diagnosing multicollinearity		Cohen’s *d*
						Tolerance	VIF	
A	Constanta	0.417		4.699	0.000			
	x_1_ Personal values	0.236	0.247	5.944	0.000	0.385	2.599	0.466
	X_2_ Attitudes towards religious organization	0.434	0.423	9.614	0.000	0.344	2.910	0.754
	x_3_ Awareness of the availability of religious counseling	0.177	0.168	4.952	0.000	0.581	1.722	0.388
B	Constanta	0.510		4.769	0.000			
	x_1_ Personal values	0.233	0.260	5.219	0.000	0.390	2.562	0.537
	x_2_ Attitudes toward religious organization	0.411	0.421	7.908	0.000	0.341	2.930	0.801
	x_3_ Awareness of the availability of religious counseling	0.205	0.204	5.018	0.000	0.585	1.711	0.508
C	Constanta	0.750		4.540	0.000			
	x_1_ Personal values	0.120	0.127	1.572	0.018	0.353	2.835	0.229
	x_2_ Attitudes toward religious organization	0.526	0.535	5.896	0.000	0.281	3.553	0.860
	x_3_ Awareness of the availability of religious counseling	0.158	0.163	2.610	0.010	0.595	1.680	0.381

Table 5 shows that the multiple regression equation model *y* = a + b1x1 + b2x2 + b3x3 + e describes the variables well. The regression model can be considered statistically significant and appropriate for all three groups of experiences and can be used further, as the coefficients of determination for groups A, B and C are *R*^2^A = 0.569; *R*^2^B = 0.625; *R*^2^C = 0.571; and the adjusted coefficients *R*^2^A = 0.567; *R*^2^B = 0.622; *R*^2^C = 0.564. In addition, the correlation analysis showed a strong connection between motives for seeking religious help and attitudes toward the religious organization, especially in Group B (ρ = 0.752). The calculated *f^2^* effect size values for every group indicate high explanatory power: *f^2^* values are 1.320 for group A, 1.667 for group B, and 1.331 for group C, which means that the model explains a very large proportion of variance in every group.

Cohen’s *d* effect sizes have shown that ‘attitudes toward religious organization’ had the strongest influence on motives for seeking religious help in all groups (Cohen’s *d* values exceed 0.75), especially in groups B and C, where *d* reached 0.801 and 0.860. This is in contrast to personal values, which had a medium-sized effect in groups A and B (Cohen’s *d* = 0.466 and 0.537), while in group C, this relationship was weak (Cohen’s *d* = 0.229). Finally, awareness of the availability of religious help had a small to medium-sized effect in all groups, with d values ranging from 0.381 to 0.508. This shows that personal values and knowledge of the availability of help have certain influence on help-seeking, but this influence is smaller compared to attitudes toward the religious organization.

The F-criterion tests whether there is at least one regressor in the estimated model on which the *motives for seeking religious counseling* depend (as the dependent variable). The results of the F-criterion are presented in the table by group: FA = 284.699; FB = 215.322; FC = 82.038; and *p* value of 0.0001 (*p* < 0.001) for all groups. The table below provides basic information on the coefficients of the regression model. The values of the standardized coefficients indicate which regressor is more influential in the model, which in this case signals the influence of *attitudes toward religious organizations* (x2) across all three groups. Moreover, *p* values explain the statistical significance of the influence of each independent variable on the dependent variable.

The regression analysis results revealed that the dimension *Awareness of access to religious counseling* (x3) *in* Groups A and B (βA = 0.168; βB = 0.204) and the dimension *Personal values* (x1) in Group C (βC = 0.127) were less influential but statistically significant as regressors, pA = 0.0001; pB = 0.0001 and pC = 0.018, respectively.

A multicollinearity problem exists when the VIF > 4.00 and the tolerance is less than 0.25. It is concluded that there is no multicollinearity problem in the specific regression model, taking into account the individual experience groups. The model was also checked for statistical outliers: Cook’s distance and DFB were calculated for each regressor observation. Cook’s distance was found to be 0.051 in group A, 0.091 in group B and 0.259 in group C, which is significantly less than unity. The maximum value of the DFB for each group of experiences of hurtful behavior by coworkers varies from 0.01127 to 0.03492 in Group A, from 0.01391 to 0.03857 in Group B, and from 0.02088 to 0.08527 in Group C and is below unity in all the regressors, suggesting that there are no outliers in the data. The normality of the data was tested via the Kolmogorov–Smirnov criterion, *p* = 0.056 in group A, *p* = 0.060 in group B, and *p* = 0.077 in group C (*p* > 0.05), and the results are consistent with the normality assumption. Residual error plots (histograms, normal P–P plots, scatter plots) show that the assumptions of normality and heteroskedasticity of the model are satisfied. The Durbin–Watson index is 1.944 in Group A, 1.946 in Group B and 2.186 in Group C, which is not significantly different from those of the other two groups, suggesting that there is no autocorrelation. Thus, three equations were constructed for the experience groups:

1. A *Reasons for seeking religious counseling* = 0.417 + 0.236 x_1_ + 0.434 x_2_ + 0.177 x_3_;
2. B *Reasons for seeking religious counseling* = 0.510 + 0.233 x_1_ + 0.411 x_2_ + 0.205 x_3_;
3. C *Reasons for seeking religious counseling* = 0.750 + 0.120 x_1_ + 0.526 x_2_ + 0.158 x_3_.

The equations show that in all three groups, attitude toward a religious organization (x2) is the strongest factor influencing motives for seeking religious counseling. Moreover, knowledge of the availability of a religious counselor (x3) has a moderate influence in all groups, whereas personal values (x1) have the lowest influence in groups B and C but are slightly stronger in group A. The constant values show that the baseline values of the motives for seeking religious counseling are different in each group, reflecting the different baseline characteristics of the groups’ experiences.

## 5 Discussion

Some recent studies suggest that religiosity is an essential resource for coping with loneliness, repetitive thinking about work issues (Azeem et al., 2024), negative emotions (De Clercq et al., 2024) and stress (Apergis et al., 2024). All of these issues are closely related to the consequences of workplace bullying (Baillien et al., 2017; Van Heugten, 2013), but religious counseling support and the factors promoting or inhibiting its seeking remain poorly researched. Therefore, this exploratory study investigated the factors influencing the decisions of workplace bullying targets to seek help from religious counselors.

The first objective was to determine what strategies religious employees choose to respond to the hostile behavior of coworkers (Q1).

Some of the main strategies–independent problem-solving (30.7%), seeking help from close family (18.5%) and passive behavior (14.1%) – are consistent with previous studies. For example, Salin et al. (2014) have found that 29.6% of respondents tried to solve problems independently, which almost coincides with our results. This suggests that independent problem-solving is a common response to workplace bullying. Interestingly, the study by Salin et al. was conducted in a different (North American) cultural context, without distinguishing religiosity.

Similarly, in another study (UK and Ireland), seeking social support was the most common strategy (Irwin et al., 2022), which is in line with the results of our research. On the other hand, the results of our study do not match the trends in help-seeking among managers and co-workers, found by Nielsen et al. (2020) in Norway. In our study, such support strategy was chosen quite rarely. In addition, other studies identified a significantly higher proportion of passively responding (avoidant) respondents. For example, in the study by Salin et al. (2014), avoidance was chosen by 54.2%. Similar trends were reported still earlier by Hoel and Cooper (2000) (United Kingdom).

The fact that results may not coincide across countries due to cultural differences is also highlighted by Nielsen et al. (2020). In addition, the role of the religious context should also be taken into account. That is, religious employees may be more inclined to trust people closest to them, especially those who share the same religious values. Furthermore, although Branch et al. (2013) have noted that if targets feel powerless, they are unlikely to seek support, the influence of religiosity cannot be ruled out either. Some research on religious coping suggest that religiosity can promote self-confidence, because individuals feel spiritual support and strength in their faith (e.g., Ano and Vasconcelles, 2005; Pargament et al., 1998). Of course, such explanation is rather speculative, but results are quite unexpected and interesting to further deepen into the role of the religious context in the help-seeking process of workplace bullying targets.

Furthermore, according to the authors, how others may perceive help-seeking is important (e.g., Kline and Lewis, 2019; O’Donnell and MacIntosh, 2016). In line with the explanations given by the respondents in this study, religious faith and shared values may have been factors that encouraged people to turn to their relatives for help in the context of the TPB and avoid seeking help in the work environment. In an environment where similar values are shared, better mutual understanding and acceptance are expected.

Several trends in religious coping have also emerged: individual, based on self-reliant religious resources; socioreligious, where a source of social support is sought in a religious community setting; and combined, where the help of a religious community professional is used in a supportive manner alongside psychological and managerial support. This confirms the trends of other studies showing that religious individuals are more likely to seek help in a religious community and that a religious counselor is seen as an essential source of help (Chaudhry and Cattaneo, 2023; Postmus et al., 2009).

The answer to the question of how different experiences of relationships with coworkers relate to motives for seeking religious counseling (Q2) was twofold. First, no reliable direct link was found between seeking religious counseling and the hostile behavior of a coworker (both occasional assault and workplace bullying). That is, motives are not directly related to situational factors. Second, these motives are most strongly (more strongly than values and knowledge of the possibilities of help) related to the person’s attitude toward the religious organization (subjective norms). This aligns with the results of Tomczyk et al. (2020), who showed that subjective norms and attitudes were positively related to help-seeking intentions (German community sample). However, their study did not consider religiosity.

On the other hand, considering the respondents’ religious identity, its influence cannot be ruled out. When persons strongly identify themselves with a group (Social Identity Theory), factors such as personal control or external situations become less important in predicting behavior (e.g., Terry et al., 1999). Therefore, the individual’s identification with the religious group may be an additional predictor of behavior in the TPB context (Iranmanesh et al., 2020). According to Willis et al. (2020), significant social identities can lead to the availability of beliefs that influence how the individual perceives norms, establishes approaches and decides on behaviors, as defined by the TPB. When individuals belong to a significant social group, a variable such as subjective norms can be directly influenced by the norms of that group. Therefore, individuals who strongly identify themselves with the religious community may ground their decision (e.g., to seek religious spiritual counseling) on the group’s norms regardless of situational factors. This suggests that representatives of the religious community, who are approached for help, should consider this perspective as well as evaluate the possibility of offering other resources of help, which may not have been considered by the religious target of workplace bullying.

Finally, we investigated how different factors influence the motives for seeking religious counseling (Q3). Attitudes toward a religious organization are directly related to subjective norms. If the affected worker believes that the religious organization is respected and that its opinion is essential, this may increase the intention to seek religious counseling. In all three variations of the regression model (A, B, C), this variable has the strongest positive effect (Table 5), indicating that attitudes toward the religious organization are among the most important factors in seeking religious counseling (especially in the group of those who have been subject to workplace bullying). Interestingly, in the group of those who have not experienced hostile behavior from coworkers (C), attitude toward the religious organization is also the most important factor in determining the motives for seeking religious counseling. That is, it is more reflective of a preconceived attitude (what would be done if respondents were attacked) and further confirms that attitudes are more important than situational factors.

In this context, it is possible to compare how research results correspond to the theoretical model of workplace bullying, proposed by Branch et al. (2013). The authors grounded on the Affective Events Theory, which states that people often react emotionally to incidents and that their reactions influence their subsequent behavior, attitudes, and ultimate wellbeing. This helps to explain how bullying acts can shape both behavioral patterns and attitudes to situations such as workplace conflicts. However, our study shows a different result, namely that participants’ attitudes toward the religious organization had a greater influence on their willingness to seek help than did the situational bullying acts. Religious individuals who felt safe in the religious organization and believed they could receive support there without being judged negatively were more likely to turn to the religious organization for help. This shows that their decision was more determined by their preconceived attitudes toward the organization than by the nature or intensity of the bullying they experienced. If the employee feels that the religious organization provides a safe space where he or she can receive support without fear of being judged or negatively evaluated, in the context of TPB, psychological safety acts as a catalyst for behaving according to one’s attitudes and norms, because in a safe environment individuals feel comfortable and can be themselves (Stühlinger et al., 2021).

Moreover, knowledge of the availability of a religious counselor is related to perceived behavioral control because knowledge of the availability of help increases individuals’ confidence that they can successfully perform a specific action (in this case, seeking religious counseling). This variable also had a positive effect on all the model variations, but its influence was somewhat smaller than that of the other variables. This suggests that awareness of opportunities is important but not the most important factor.

### 5.1 Practical implications

The results of this study can contribute to a better understanding of how religiosity and the religious community’s support can be useful in providing support to employees facing workplace bullying. First, since the attitude toward the religious organization in the investigated population is one of the most important factors determining the decision to seek help, religious communities should promote support for workplace bullying targets as an important norm of the community, simultaneously creating a psychologically safe and supportive environment. This can be done through community gatherings, events where emotional problems are discussed, including workplace bullying.

Second, religious communities can publicize success stories where religious support has benefited victims of workplace bullying. This would help community members to form a positive opinion about the effectiveness and significance of consultations.

Third, because knowledge of the availability of religious counselors promotes people to seek help, the development of this area may be beneficial in seeking to increase perceived behavioral control. This can be done by organizing information campaigns about availability of counseling opportunities. Finally, specialized training for community leaders and religious counselors would be useful to help them better understand the factors motivating workplace victims to seek help and what kind of help could be provided to them.

Thus, religious communities can actively contribute to reducing the psychological impact of workplace bullying by providing social, emotional and religious spiritual support. Religious communities can mediate between victims and assitance resources, such as psychological or social support, this way removing some of the barriers hindering timely help-seeking, such as the lack of information about support options, fear of condemnation or the lack of understanding. Therefore, leaders of religious organizations should not only encourage openness and support but also inform members about specific help options, which would help reduce delays in seeking help during workplace conflicts. It is likely that this will reduce victims’ isolation and speed up the provision of support, thus reducing the negative effects of workplace bullying.

### 5.2 Limitations of the study and further research

The main limitations are related to the limited ability to compare the study results with those of the study design. The impossibility of making a clear distinction between cause and effect in this type of research, the confounding effect of confounding variables, and the risks of respondents’ memory (respondents may have inaccurately recalled certain incidents they experienced). Although the confounding effect cannot be completely avoided, to reduce their influence, statistical methods were used. For example, the regression analysis helped to identify the most important variables that had the strongest impact on the research results, which helps to highlight possible trends even in the presence of certain unexplained factors. Several procedural measures, recommended by Podsakoff et al. (2003), to reduce bias were also applied. Specifically, neutral statements were formulated seeking to avoid social desirability bias, statements were arranged in a random order, and anonymity was ensured. There were opportunities to fill in questionnaires in a safe environment chosen by respondents themselves, without limiting the time required for completing the questionnaire and guaranteeing anonymity and confidentiality. This allowed respondents to better recall personal experiences without additional stress. In addition, through the mediation of community leaders, larger and more diverse groups of respondents were reached, which might otherwise be difficult to reach. This reduced dominance of one group or community in the sample, increasing the representativeness of results. Respondents from different regions of the country were also reached through religious community networks, thus reducing geographical limitations.

The study results broadly reflect the Catholic context, and the sampling method chosen does not guarantee representativeness; however, it should also be appreciated that this study is exploratory and intended to lay the groundwork for further research. In future studies, it would make sense to repeat the study in different religious contexts, combined with an extended qualitative study and additional assessment of demographic variables.

Although representatives of other religions may have different experiences, influenced by different religious norms and traditions, this study highlights psychological safety, when the individual feels that he/she can seek help without being judged negatively. Such approach may be universal and applicable to other religious settings, since psychological safety within a religious community can act as an important factor, regardless of faith tradition. However, future research involving representatives of other religious denominations could help identify the extent to which these findings can be generalized and how religious communities could contribute to enhancing psychological safety, despite faith differences.

Since changes in external factors and context over time were not considered, a longitudinal study would be useful in the future. This would allow to better assess changes in employees’ attitudes toward the religious organization and help-seeking behavior. More attention could also be paid to the relation of demographic factors (e.g., age, gender, occupation) to help-seeking behavior. The analysis of these factors would show how different groups respond to workplace bullying and how religious support can be applied to different populations. Since the results of this study have shown that in practice religious spiritual help is combined with psychological help and support in the organization, it would make sense to examine in more depth the effects of different types of help or their combination. This study did not examine the efficiency of religious counseling, and future studies could also compare the help-seeking motives among religious and non-religious employees as well as interactions between religious and non-religious help systems.

This study focused on the role of religious communities and motives determining help-seeking. Although psychological reactions to bullying, such as stress or depression, can be important, they were not directly analyzed within the scope of this study. In the future, research could include a more detailed analysis of psychological factors such as stress, anxiety or self-stigmatization and their impact on religious or other types of help-seeking. It would also be useful to compare how different cultural environments affect help-seeking behavior and perceptions of bullying. In addition, it would be beneficial to conduct a more exhaustive research on the ways in which different religious institutions provide support and their impact on victims in the workplace.

## Data Availability

The raw data supporting the conclusions of this article will be made available by the authors, without undue reservation.

## Footnotes

^1^ https://uzt.lt/en

^2^ https://osp.stat.gov.lt/informaciniai-pranesimai?eventId=288049
